# The Heterodimeric ABC Transporter EfrCD Mediates Multidrug Efflux in Enterococcus faecalis

**DOI:** 10.1128/AAC.00661-16

**Published:** 2016-08-22

**Authors:** Lea M. Hürlimann, Valentina Corradi, Michael Hohl, Guido V. Bloemberg, D. Peter Tieleman, Markus A. Seeger

**Affiliations:** a Institute of Medical Microbiology, University of Zurich, Zurich, Switzerland; b Centre for Molecular Simulation and Department of Biological Sciences, University of Calgary, Calgary, Alberta, Canada

## Abstract

Nosocomial infections with Enterococcus faecalis are an emerging health problem. However, drug efflux pumps contributing to intrinsic drug resistance are poorly studied in this Gram-positive pathogen. In this study, we functionally investigated seven heterodimeric ABC transporters of E. faecalis that are annotated as drug efflux pumps. Deletion of *ef0789-ef0790* on the chromosome of E. faecalis resulted in increased susceptibility to daunorubicin, doxorubicin, ethidium, and Hoechst 33342, and the corresponding transporter was named EfrCD. Unexpectedly, the previously described heterodimeric multidrug ABC transporter EfrAB contributes marginally to drug efflux in the endogenous context of E. faecalis. In contrast, heterologous expression in Lactococcus lactis revealed that EfrAB, EfrCD, and the product of *ef2226-ef2227* (EfrEF) mediate the efflux of fluorescent substrates and confer resistance to multiple dyes and drugs, including fluoroquinolones. Four of seven transporters failed to exhibit drug efflux activity for the set of drugs and dyes tested, even upon overexpression in L. lactis. Since all seven transporters were purified as heterodimers after overexpression in L. lactis, a lack of drug efflux activity is not attributed to poor expression or protein aggregation. Reconstitution of the purified multidrug transporters EfrAB, EfrCD, and EfrEF in proteoliposomes revealed functional coupling between ATP hydrolysis and drug binding. Our analysis creates an experimental basis for the accurate prediction of drug efflux transporters and indicates that many annotated multidrug efflux pumps might be incapable of drug transport and thus might fulfill other physiological functions in the cell.

## INTRODUCTION

The Gram-positive bacterium Enterococcus faecalis is a normal inhabitant of the human gastrointestinal tract (1) and generally displays low levels of virulence (2). E. faecalis is a facultatively anaerobic coccus that survives under extreme environmental conditions, including extreme pH and temperature ranges. It frequently acquires antibiotic resistance via horizontal gene transfer (3). These traits have led to its emergence as a major nosocomial pathogen associated with serious diseases, such as bacteremia, endocarditis, urinary tract infections, and surgical wound infections, which are difficult to treat with antibiotics (4). Whole-genome sequencing of E. faecalis V583, a vancomycin-resistant clinical isolate, has revealed that more than one-quarter of the predicted protein-encoding open reading frames (ORFs) originate from mobile and exogenously acquired DNA (5). Among the transferred genes are so-called Van clusters, which confer resistance to the clinically important antibiotic vancomycin, used to treat β-lactam-resistant E. faecalis (3).

While the mechanisms underlying resistance to β-lactams, aminoglycosides, fluoroquinolones, and vancomycin are well documented, comparatively little is known about drug efflux pumps in E. faecalis. The genome of E. faecalis V583 contains 34 genes encoding potential multidrug resistance (MDR) pumps belonging to four transporter superfamilies (6). However, their contribution to intrinsic resistance against antibiotics is poorly studied. In contrast to the closely related genus Enterococcus faecium, E. faecalis is naturally resistant to quinupristin-dalfopristin, a drug mixture targeting the ribosome, which was developed to treat vancomycin-resistant enterococci (7). Quinupristin-dalfopristin resistance had been linked to the *lsa* (*ef2720*) gene, which is present in E. faecalis but absent from E. faecium (8). Disruption of *lsa* in E. faecalis results in >40-fold-increased susceptibility to quinupristin-dalfopristin. This gene encodes two fused nucleotide binding domains (NBDs), which are typically part of ABC transporters. However, no open reading frame encoding an ABC transporter transmembrane domain (TMD), which could work in concert with Lsa to constitute a drug efflux pump, has been identified thus far. Recently, the Lsa homologue OptrA—encoded on a large transferable plasmid—was reported to confer resistance to linezolid in enterococci (9). In analogy to Lsa, no transmembrane domain belonging to an ABC transporter was found to be encoded on the plasmid. A very elegant recent study finally revealed that Lsa and OptrA belong to the ABC-F subfamily of ATP-binding cassette proteins, which protect the ribosome from the noxious effect of antibiotics by displacing the drugs from their target binding sites (10). Further, the major facilitator superfamily transporter EmeA (EF1078), a close homologue of the well-characterized MDR transporter NorA of Staphylococcus aureus, has been shown, on the basis of experiments performed with a corresponding transporter gene deletion, to mediate basal resistance to ethidium and norfloxacin in E. faecalis (11). Finally, the heterodimeric ABC transporter EfrAB (EF2920–EF2919 [EF2920/19]) has been proposed to be an MDR pump transporting norfloxacin and acriflavine when overexpressed in Escherichia coli (12), but its functional role in E. faecalis was not experimentally studied by a respective gene deletion.

ABC exporters are a subclass of ABC transporters found in all living cells. They are composed of at least four domains: two TMDs and two NBDs. Bacterial ABC exporters are encoded as half-transporters containing a TMD fused to an NBD, which form either homodimers, upon the dimerization of two identical polypeptides (e.g., Sav1866, MsbA), or heterodimers, from two different polypeptides (e.g., LmrCD, PatAB, TM287–TM288 [TM287/288]). In contrast, most eukaryotic ABC exporters are encoded on a single large polypeptide chain (e.g., P-gp, MRP1, CFTR, SUR1). The architecture of ABC exporters has been characterized by crystal structures of the homodimers Sav1866 (13), MsbA (14), CmABCB1 (15), ABCB10 (16), and McjD (17) and the heterodimers P-gp (ABCB1, MDR1) (18, 19) and TM287/288 (20, 21). The 12 transmembrane helices, 6 from each TMD, are responsible for substrate recognition and form a substrate pathway across cellular membranes by alternating between inward- and outward-oriented states. Conformational transitions between the states is driven by the binding and hydrolysis of ATP at the NBDs and is transferred to the TMDs via intracellular loops. The two NBDs are arranged in a head-to-tail conformation, leading to the formation of two ATP binding sites at their interface. Homodimeric ABC transporters consist of two identical ATP binding sites, whereas many heterodimeric ABC transporters exhibit asymmetric ATP binding sites. In these heterodimeric ABC transporters, one nucleotide binding site, called the degenerate site, deviates in the residues responsible for ATP hydrolysis but is still able to bind nucleotides tightly (22). The second ATP binding site is canonical and is thus called the consensus site (20).

Several heterodimeric ABC exporters have been reported to be involved in multidrug efflux in Gram-positive bacteria. These include Lactococcus lactis LmrCD (23) and Streptococcus pneumoniae PatAB (24, 25). Here we studied seven E. faecalis homologues of LmrCD and PatAB with regard to drug efflux. Among these seven transporters, EfrAB was the only one that had been characterized previously as a multidrug efflux pump when expressed in E. coli (see above) (12).

Functional studies using unmarked gene deletions of the seven transporters revealed that *ef0789*–*ef0790* is the major heterodimeric multidrug ABC exporter of E. faecalis, and its gene product was dubbed EfrCD. Chromosomal deletion of *efrAB* revealed modest susceptibility to acriflavine and ethidium, and overexpression of *efrAB* in L. lactis confirmed its activity as a multidrug efflux pump. Finally, the gene product of *ef2226*–*ef2227* was identified as another novel drug efflux pump and was called EfrEF.

## MATERIALS AND METHODS

### Bacterial strains, growth media, and chemicals.

Enterococcus faecalis V583 (ATCC 700802) was obtained directly from the American Type Culture Collection (ATCC). Enterococcus faecalis 4205 is a chloramphenicol- and erythromycin-sensitive clinical isolate obtained from the diagnostic unit of the Institute of Medical Microbiology, University of Zurich. The sequence of its 16S rRNA locus has been deposited in GenBank under accession number KU936078. The 16S rRNA sequence of E. faecalis 4205 is identical to that of E. faecalis ATCC 29212 (no mismatches in 1,468 bp). The closest 16S rRNA sequence not belonging to the species E. faecalis is that of Enterococcus rivorum (10 mismatches in 1,468 bp), showing that species determination was unambiguous. E. faecalis strains V583 and 4205 were grown in half-strength brain heart infusion (hBHI; Bacto) at 30°C or 42°C for routine maintenance as well as in cation-supplemented Mueller-Hinton broth (MHB) at 37°C for MIC determination. M9 salts supplemented with 0.25% yeast extract and 0.5% glucose (MM9YEG) was used for the generation of gene deletions (26). L. lactis NZ9000 Δ*lmrA* Δ*lmrCD* was grown in M17 supplemented with 0.5% glucose (GM17; Oxoid) at 30°C without shaking. E. coli strains MC1061 and XL1-Blue were grown in Luria broth (LB) at 30°C if they contained plasmids with a temperature-sensitive origin of replication and at 37°C otherwise. The concentrations of antibiotics used for plasmid propagation were as follows: chloramphenicol, 20 μg/ml (E. faecalis), 5 μg/ml (L. lactis), or 20 to 25 μg/ml (E. coli); ampicillin, 100 to 120 μg/ml (E. coli); erythromycin, 10 μg/ml (E. faecalis) or 5 μg/ml (L. lactis). All chemicals and antibiotics were purchased from Sigma-Aldrich.

### Homology search using protein BLAST.

The six half-transporters LmrC (CAL96930), LmrD (CAL96931), PatA (NP_359478), PatB (NP_359476), TM287 (Q9WYC3), and TM288 (Q9WYC4) were used as query sequences to search for heterodimeric ABC transporters in Enterococcus faecalis V583 (taxid 226185) using protein BLAST. Protein hits with an expect value (E value) smaller than 1E−4 were considered homologues. Furthermore, we restricted our search to heterodimeric transporters, which are encoded on two adjacent open reading frames harboring a transmembrane domain (TMD) and a nucleotide binding domain (NBD) and containing a degenerate and a consensus ATP binding site.

### Construction of E. faecalis gene deletion mutants.

Gene deletions were generated using the counterselection strategy based on a dominant negative mutant of the thymidylate synthase gene, thyA*, in a two-step recombination mechanism (27). Flanking regions of the genes of interest (see Fig. 2) were amplified from E. faecalis 4205 and E. faecalis V583 using primers (see Table S2 in the supplemental material) designed on the basis of the genome sequence of E. faecalis V583. All primers worked equally well for E. faecalis 4205, confirming the close sequence relationship between E. faecalis 4205 and E. faecalis V583 observed for the 16S rRNA sequence. The two flanking regions were fused by overlapping PCR and were cloned into the gene deletion vector pCJK245_FX using fragment exchange (FX) cloning (28). To create the FX-cloning-compatible gene deletion vector pCJK245_FX, a fragment containing the *ccdB* marker and the two SapI restriction sites was amplified from pINIT_cat (28) with primers containing an XbaI or NcoI restriction site as an overhang (see Table S3 in the supplemental material) and was cloned into pCJK245 (27). The gene deletion vector pCJK245_FX contains a temperature-sensitive origin of replication, which permits plasmid replication at 30°C but not at 42°C. The gene deletion vectors were transformed into electrocompetent E. faecalis cells and were selected for resistance to chloramphenicol at 30°C. Colonies were grown in hBHI in the presence of chloramphenicol at 30°C overnight. This preculture was diluted 1:100 into fresh medium and was grown at 30°C for 3 h, followed by a temperature shift to 42°C for 2 h to facilitate the first recombination (integration). Cells were plated onto hBHI agar plates containing chloramphenicol and 5-bromo-4-chloro-3-indolyl-β-d-galactopyranoside (X-Gal) (120 μg/ml) and were grown at a nonpermissive temperature (42°C). In order to facilitate the second recombination step, blue colonies of the first recombinants were picked and were propagated in thymine-poor MM9YEG medium in the absence of chloramphenicol. In this medium, no growth was expected for cells containing the gene deletion vector due to the dominant negative counterselection marker ThyA*, which dimerizes with wild-type ThyA and blocks its capability to synthesize thymine. However, counterselection strength was limited, i.e., all first recombinants grew readily in MM9YEG. Counterselection strength could not be improved by using a chemically defined medium completely lacking thymine. Therefore, the first recombinants were screened by monitoring cell growth (expressed as the optical density at 600 nm [OD_600_]) to identify those that grew comparatively slowly in MM9YEG. Slow-growing clones (20% of 1,000 clones tested) were streaked out onto MM9YEG agar plates containing X-Gal (120 μg/ml) and were incubated at 30°C. White colonies (in total 600 for the seven deletion mutants) were screened for gene deletions using colony PCR (with GoTaq G2 DNA polymerase; Promega). Genomic DNA was prepared from chloramphenicol-sensitive clones, and PCR amplifications were performed to confirm gene deletions (Phusion DNA polymerase; Thermo Fisher Scientific). Although a ∼1:1 ratio was expected between mutant and wild-type strains as a result of the second recombination, we obtained only 5% gene deletions, whereas 95% of strains reverted to wild type. Finally, we were able to generate markerless gene deletions for the seven heterodimeric ABC exporters in E. faecalis strain 4205 and to delete the *efrAB* and *efrCD* transporters from the genome of the vancomycin-resistant E. faecalis strain V583 (see Fig. 2).

### MIC determination.

The MICs of antibiotics were determined by broth dilution in microtiter plates. MICs for E. faecalis were determined according to EUCAST guidelines (29). E. faecalis was grown to saturation overnight in cation-supplemented Mueller-Hinton broth (MHB), diluted 1:100 in fresh medium, and grown at 37°C for 3 h to reach a cell density equal to or greater than a 0.5 McFarland standard. The microtiter plates containing 2-fold serial dilutions of antibiotics in cation-supplemented MHB were inoculated with 5 × 10^5^ CFU/ml. The plates were incubated at 37°C for 18 h. MICs for L. lactis were determined as described in previous studies (20, 30). L. lactis cells were grown in GM17 at 30°C to saturation overnight, diluted 1:100 in fresh medium, and then grown at 30°C for 3.5 h to reach the exponential-growth phase. These cells were used to inoculate (1:100) microtiter plates containing drugs and nisin (a nisin-containing culture supernatant of L. lactis NZ9700 was added to the medium at 1:5,000 [vol/vol]). Inoculated plates were incubated at 30°C for 16 h. The lowest antibiotic concentration preventing growth was determined to be the MIC.

### Complementation of gene deletion mutants.

E. faecalis V583 Δ*efrCD* and E. faecalis 4205 Δ*efrCD* were complemented with a plasmid expressing wild-type *efrCD*. As a control, EfrCD was inactivated by the introduction of an E-to-Q mutation in the consensus site. The ORFs of *efrCD* were amplified with its endogenous promoter (200 bp upstream of the start codon) (see Table S4 in the supplemental material) and were cloned into plasmid pMSP3535_FX harboring either an erythromycin (for E. faecalis 4205) or a chloramphenicol (for E. faecalis V583) resistance marker (31). These two complementation vectors were modified from pMSP3535 (catalog no. 46886; Addgene). To generate pMSP3535_FX_em, the pMSP3535 vector was amplified with primers containing SapI restriction sites as overhangs (Table S3 in the supplemental material). This PCR product was cut with SapI and was ligated with the fragment containing the *ccdB* marker and the chloramphenicol resistance gene obtained by SapI digestion of pREXNH3 (28). Since E. faecalis V583 is resistant to erythromycin, the resistance gene of pMSP3535_FX_em was exchanged with a chloramphenicol resistance gene. To this end, pMSP3535_FX_em was amplified with 5′-phosphorylated primers and was ligated via blunt ends with the chloramphenicol resistance gene that was amplified from pCJK245. The chloramphenicol resistance gene was oriented in the same direction as the erythromycin resistance gene in pMSP3535_FX_em. The MICs of the complemented strains were determined as described above in cation-supplemented MHB containing erythromycin or chloramphenicol to propagate the plasmids.

### Assay for the accumulation of fluorescent dyes.

L. lactis NZ9000 Δ*lmrA* Δ*lmrCD* cells harboring plasmids encoding the seven transporters (in their wild-type or inactive E-to-Q mutant forms) were grown in GM17 containing 5 μg/ml chloramphenicol at 30°C. Expression was induced at an OD_600_ of 0.4 to 0.6 with a nisin-containing culture supernatant of L. lactis NZ9700 for 1 h (1:1,000 [vol/vol]). Cells were washed and were resuspended with fluorescence buffer (50 mM KP_i_ at pH 7.0, 5 mM MgSO_4_). Cells were adjusted to an OD_600_ of 0.5 in 2 ml fluorescence buffer and were energized by adding 0.5% glucose. Nigericin and valinomycin (1 μM each) were added prior to the addition of 2′,7′-bis-(2-carboxyethyl)-5(6)-carboxyfluorescein acetoxymethyl ester (BCECF-AM) to avoid changes in BCECF fluorescence as a result of pH changes in the cytoplasm. The accumulation of 5 μM ethidium, 0.5 μM Hoechst 33342, or 0.2 μM BCECF-AM was monitored at 25°C for 600 s using an LS-55 fluorescence spectrometer (PerkinElmer). Excitation and emission wavelengths (with slit widths given in parentheses) were set at 520 nm (10 nm) and 595 nm (15 nm) for ethidium, 355 nm (5 nm) and 457 nm (5 nm) for Hoechst 33342, and 502 nm (2.5 nm) and 525 nm (4.0 nm) for BCECF, respectively (see Fig. 3 and Fig. S1 in the supplemental material).

### Cloning, expression, and purification of transporters in L. lactis.

Enterococcal ORFs were amplified from the genomic DNA of E. faecalis V583 (Table S4 in the supplemental material), cloned into the pREXNH3 shuttle vector by FX cloning (28), and then subcloned into the pNZ8048NH3 expression vector via vector-backbone exchange (VBEx) cloning (32). The inactive E-to-Q mutant was generated by mutating the conserved Walker B glutamate of the consensus site to a glutamine using QuikChange site-directed mutagenesis (primer sequences are given in Table S4). Expression vectors harboring the gene of interest were transformed into electrocompetent L. lactis NZ9000 Δ*lmrA* Δ*lmrCD* cells (33). The cells were grown in GM17 and 5 μg/ml chloramphenicol at 30°C to an OD_600_ of 1 and were then induced by adding a nisin-containing culture supernatant of L. lactis NZ9700 for 4 h (1:5,000 [vol/vol]). Membranes were prepared by disrupting the cells with a Microfluidizer processor (Microfluidics) at 30,000 lb/in^2^ in phosphate-buffered saline (PBS) buffer (pH 7.4) containing 15 mM K-EDTA (pH 7.4) and a protease inhibitor. The supernatant from low-spin centrifugation (8,000 × *g*, 10 min, 4°C) was supplemented with 30 mM MgCl_2_ and DNase I (1:1,000 [vol/vol]) and was incubated for 30 min at 4°C. Membranes were collected by high-spin centrifugation (38,000 rpm, 1 h, 4°C; 45 Ti rotor; Beckman) and were resuspended in Tris-buffered saline (TBS) (pH 7.5) containing 10% glycerol. Membranes were solubilized for 2 h using 1% (wt/vol) *n*-dodecyl-β-d-maltoside (β-DDM), and unsolubilized material was removed by high-spin centrifugation. The resulting supernatant was loaded onto a Ni^2+^-nitrilotriacetic acid (NTA) column, washed with 50 mM imidazole (pH 7.5), 200 mM NaCl, 10% glycerol, and 0.03% β-DDM, and eluted with 200 mM imidazole (pH 7.5), 200 mM NaCl, 10% glycerol, and 0.03% β-DDM. Protein dialysis against 20 mM Tris-HCl (pH 7.4), 150 mM NaCl, and 0.03% β-DDM and cleavage of the deca-His tag with 3C protease were performed simultaneously overnight. The deca-His tag and the His-tagged 3C protease were removed by Ni^2+^-NTA chromatography. The cleaved protein was analyzed by size exclusion chromatography (SEC) on a Superdex 200 Increase 10/300 GL column in 20 mM Tris-HCl (pH 7.4), 150 mM NaCl, and 0.03% β-DDM. The protein concentration was determined by measuring *A*_280_ using a NanoDrop 2000c spectrophotometer.

### Reconstitution into E. coli polar lipids.

E. coli total lipids (Avanti Polar Lipids) were dissolved in chloroform, washed by adding acetone while stirring at 4°C overnight, and harvested by centrifugation in glass vials (3,000 × *g*, 10 min, 4°C). E. coli polar lipids were dissolved in diethyl ether, and nonsoluble lipids were removed by centrifugation (3,000 × *g*, 10 min, 4°C). Diethyl ether was evaporated in a rotary evaporator, and the E. coli polar lipids were dissolved in chloroform to yield 20 mg/ml. Lipid mixtures of E. coli polar lipids and l-α-phosphatidylcholine (from egg yolk; catalog no. P3556; Sigma) were prepared in chloroform at a ratio of 3:1 (wt/wt). Chloroform was removed in a rotary evaporator, and lipids were suspended in 50 mM K-HEPES (pH 7.0). The suspension was sonicated to form small unilamellar vesicles (SUVs). Thawing and flash-freezing fused the SUVs to large multilamellar vesicles (LMVs), which were extruded 11 times through a 400-nm polycarbonate filter to form large unilamellar vesicles (LUVs). LUVs were diluted to a working concentration of 4 mg/ml and were destabilized using 5.25 mM Triton X-100 (34). The detergent-purified proteins EfrAB, EfrCD, EfrEF, and EF0942–EF0941 (EF0942/41) (both wild type and E-to-Q mutants) were added to the destabilized liposomes at a protein/lipid ratio of 1:100. Four rounds of addition and removal of Bio-Beads (SM-2 polystyrene beads; Bio-Rad) were performed to remove detergent molecules. In each round, 200 mg Bio-Beads was added to the reconstitution reaction mixture. The four rounds consisted of incubation at room temperature for 30 min, at 4°C for 1 h, at 4°C overnight, and at 4°C for 1 h, respectively. Bio-Beads were removed by filtration, and proteoliposomes were harvested (40,000 rpm, 40 min, 4°C; 70 Ti rotor; Beckman). Proteoliposomes were resuspended in 50 mM K-HEPES (pH 7.0) to obtain a final concentration of 4 mg/ml lipids. Protein concentrations were determined based on the signal intensity on an SDS-PAGE gel stained with Sypro Ruby (Molecular Probes) using detergent-purified proteins as a reference. The concentrations of the detergent-purified proteins were determined by measuring *A*_280_ using a NanoDrop 2000c spectrophotometer.

### ATPase activity assay.

For the determination of basal ATPase activity, detergent-purified or reconstituted protein was mixed with 1 mM ATP and was incubated at 30°C for 15 min. A malachite green–molybdate solution was added as described previously (21). This solution forms a complex with the released inorganic phosphate and is detected colorimetrically by measuring *A*_640_. ATPase reactions for detergent-purified proteins were carried out in 20 mM Tris-HCl (pH 7.4), 150 mM NaCl, 10 mM MgSO_4_, and 0.03% β-DDM (see Table 4). For reconstituted proteins, 50 mM K-HEPES (pH 7.0) and 10 mM MgSO_4_ without the addition of β-DDM was used (see Table 4 and Fig. 5). Daunorubicin, ethidium, and Hoechst 33342 were added at final concentrations ranging from 0.1 μM to 200 μM.

### Homology modeling.

To generate the homology model of EfrCD, a multiple-sequence alignment was performed, including (i) the sequences of the seven enterococcal ABC transporters; (ii) the sequences of LmrCD and PatAB, used as references for bacterial heterodimeric ABC transporters; and (iii) the sequences of homologous bacterial transporters for which crystal structures are available, namely, TM287/288 (20), Sav1866 (13), and MsbA (14). The sequence alignment was generated using MAFFT (35). Figures S3 and S4 in the supplemental material show the final alignment for TMDs and NBDs, respectively, color coded according to the Clustal X color scheme, implemented in Jalview (36). The model of EfrCD was built with Modeler, version 9.15 (37), using the structure of TM287/288 (20) as a template. Twenty structures were generated and were sorted according to the Discrete Optimized Protein Energy (DOPE) score and root mean square deviation (RMSD) values calculated on the α-carbon only with respect to the template. Among the 10 best models, the representative model was chosen based on visual inspection of the side chain orientations of critical residues at the NBDs, i.e., the Q-loop glutamine, the switch loop histidine (or glutamine), and the catalytic residue of the Walker B motif (either aspartate or glutamate).

## RESULTS

### Identification of heterodimeric ABC exporters in Enterococcus faecalis.

**FIG 1:**
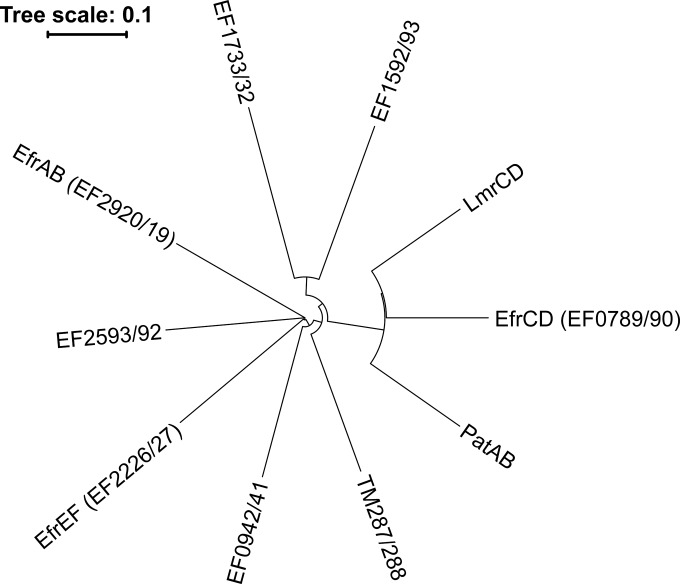
Phylogenetic tree of heterodimeric ABC transporters. Seven E. faecalis ABC transporters were aligned with Lactococcus lactis LmrCD, Streptococcus pneumoniae PatAB, and Thermotoga maritima TM287/288. All ABC transporters contain a degenerate and a consensus ATP binding site.

The genome of Enterococcus faecalis V583 was scanned for the presence of heterodimeric ABC exporters, which are potentially involved in drug efflux. Heterodimeric ABC exporters are characterized by two hallmarks, to which we confined our search. First, they are encoded on two separate genes, each encoding a half-transporter consisting of one TMD and one NBD which are adjacent to each other and in some cases even overlap on the genome. Second, most heterodimeric ABC exporters contain asymmetric NBDs with a degenerate and a consensus ATP binding site. Three well-characterized heterodimeric ABC drug exporters, Lactococcus lactis IL1403 LmrCD (23), Streptococcus pneumoniae R6 PatAB (25), and Thermotoga maritima MSB8 TM287/288 (20), served as query sequences, and each of the six half-transporters was used to conduct protein BLAST searches. Genome scanning revealed that an astonishing number of heterodimeric ABC exporters—seven—were present in E. faecalis V583, with high sequence identities of 27 to 43% to each other (Fig. 1; see also Table S1 in the supplemental material).

### Endogenously produced EfrCD mediates pronounced drug efflux.

**FIG 2:**
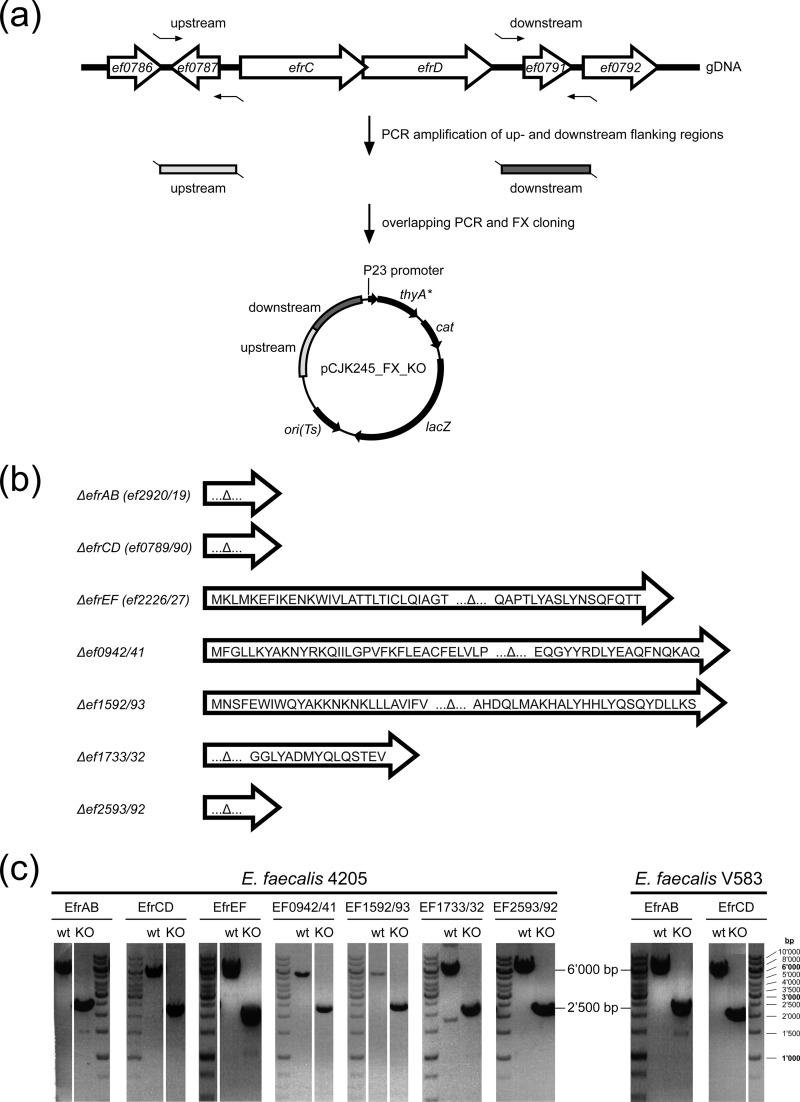
Generation of gene deletions in E. faecalis. (a) Genetic environment of a heterodimeric ABC transporter gene cluster (*efrCD* is presented as an example) in the context of upstream (*ef0786*, *ef0787*) and downstream (*ef0791*, *ef0792*) genes. Upstream and downstream flanking regions (∼1,000 bp each) were amplified from genomic DNA (gDNA) and were cloned into the gene deletion vector pCJK245_FX. KO, knockout. (b) Translational products of gene remnants after transporter gene deletion. (c) Confirmation of transporter gene deletions in E. faecalis by PCR amplification from wild-type (∼6,000 bp) and mutant (∼2,500 bp) genomic DNA with primers used to amplify the upstream and downstream regions (5′-FW and 3′-RV [see Table S2 in the supplemental material]). Gene deletions for all seven heterodimeric ABC exporters were generated in E. faecalis 4205. The two transporter genes *efrAB* and *efrCD* were also deleted in E. faecalis V583.

Unmarked gene deletions were generated by using the dominant negative mutant of the thymidylate synthase gene, thyA*, as a counterselection marker (27). Two E. faecalis strains were used: a clinical strain isolated in-house, called E. faecalis 4205, and the vancomycin-resistant E. faecalis strain V583 (5). Gene deletions were easier to generate in E. faecalis 4205 than in E. faecalis V583. Therefore, gene deletions of the seven transporters were initially made in E. faecalis 4205. Gene deletions were designed such that 95 to 100% of the open reading frames (ORFs) were deleted (Fig. 2a and b). The deletions were confirmed by PCR amplification from genomic DNA (Fig. 2c). None of the gene deletion mutants displayed any growth deficiencies (not shown).

MICs for 13 typical drug efflux pump substrates were determined in liquid culture. Sensitivities to doxorubicin, daunorubicin, Hoechst 33342, and ethidium were increased 32-, 8-, 4-, and 2-fold, respectively, when *ef0789*–*ef0790* was deleted in E. faecalis 4205 (Table 1). The gene product, EF0789/90, was therefore identified as a multidrug efflux pump in the endogenous context of E. faecalis. In analogy to EfrAB (12), the transporter was termed EfrCD (E. faecalis multidrug resistance proteins C and D). Unexpectedly, E. faecalis 4205 Δ*efrAB* had a much less pronounced phenotype than E. faecalis 4205 Δ*efrCD*, exhibiting only 2-fold-reduced resistance to acriflavine and ethidium. A 2-fold reduction in the MIC of acriflavine was also observed upon deletion of the *ef2226* and *ef2227* genes. The corresponding gene product was named EfrEF. For the other four transporter gene deletions, no changes in drug sensitivity were detectable. The *efrAB* and *efrCD* genes were also deleted in E. faecalis V583. MIC determination revealed identical MIC changes for the *efrCD* gene deletion, indicating that the dominant role of *efrCD* is observed independently of the strain. In contrast, MIC determination with E. faecalis V583 Δ*efrAB* did not confirm the 2-fold-increased susceptibilities to acriflavine and ethidium observed in the corresponding E. faecalis 4205 Δ*efrAB* strain, indicating that this transporter plays a rather limited role with regard to drug efflux in the endogenous context of different E. faecalis strains.

**TABLE 1:** MICs for E. faecalis 4205 and E. faecalis V583 gene deletion strains

Drug tested	MIC (μg/ml)^*a*^ for:										
	E. faecalis 4205								E. faecalis V583		
	Wild type	Δ*efrAB*	Δ*efrCD*	Δ*efrEF*	Δ*ef0942*-*ef0941*	Δ*ef1592*-*ef1593*	Δ*ef1733*-*ef1732*	Δ*ef2593*-*ef2592*	Wild type	Δ*efrAB*	Δ*efrCD*
Ciprofloxacin	1	1	1	1	1	1	1	1	0.5	0.5	0.5
Norfloxacin	4	4	4	4	4	4	4	4	2	2	2
Ofloxacin	2	2	2	2	2	2	2	2	1	1	1
Gentamicin	16	16	16	16	16	16	16	16	>1,024	>1,024	>1,024
Kanamycin	64	64	64	64	64	64	64	64	>1,024	>1,024	>1,024
Minocycline	16	16	16	16	16	16	16	16	0.125	0.125	0.125
Tetracycline	64	64	64	64	64	64	64	64	0.5	0.5	0.5
Rifampin	4	4	4	4	4	4	4	4	0.5	0.5	0.5
Daunorubicin	16	16	**2**	16	16	16	16	16	16	16	**2**
Doxorubicin	128	128	**4**	128	128	128	128	128	128	128	**4**
Acriflavine	16	**8**	16	**8**	16	16	16	16	16	16	16
Ethidium	16	**8**	**8**	16	16	16	16	16	16	16	**8**
Hoechst 33342	1	1	**0.25**	1	1	1	1	1	0.5	0.5	**0.125**

a MICs were determined in cation-supplemented Mueller-Hinton broth after 18 h at 37°C from a minimum of three independent experiments. Differences of ≥2-fold from the MIC for the wild type are shown in boldface.

### Complementation and overexpression of *efrCD* in E. faecalis.

E. faecalis 4205 Δ*efrCD* was complemented with a plasmid containing *efrCD* preceded by its endogenous promoter (pMSP3535_FX_em for E. faecalis 4205; pMSP3535_FX_cat for E. faecalis V583) (31). As a control, a complementation plasmid was constructed on which the transporter's consensus site Walker B glutamate was mutated to glutamine (E512Q mutation in EfrD). This mutant is not capable of ATP hydrolysis and drug transport. As expected, the mutant transporter was unable to complement E. faecalis 4205 Δ*efrCD* (Table 2). In contrast, when the gene deletion strain was complemented with the wild-type transporter, it again became resistant to daunorubicin and Hoechst 33342. The levels of resistance to daunorubicin and Hoechst 33342 were twice as high as those in wild-type E. faecalis 4205 (Table 1). We reasoned that this was due to transporter overexpression from the complementation plasmid. Indeed, when we transformed the complementation vector encoding the wild-type transporter into the original E. faecalis 4205 strain, we observed the same 2-fold increase in resistance to daunorubicin and Hoechst 33342 over that of E. faecalis 4205 that did not contain any plasmid (Tables 1 and 2). Using *efrCD* cloned into pMSP3535_FX_cat, we were able to complement E. faecalis V583 Δ*efrCD* as well (not shown). We also attempted to complement E. faecalis 4205 Δ*efrAB* with *efrAB*, either expressed from the nisin-inducible vector pMSP3535 (31) or cloned in the context of the native promoter. However, these attempts were not successful, presumably because *efrAB* was not expressed from these vectors in E. faecalis.

**TABLE 2:** Complementation of E. faecalis 4205 Δ*efrCD* with a plasmid producing wild-type EfrCD or the inactive E512Q mutant from the native promoter

E. faecalis 4205 genotype	Complementation	MIC (μg/ml)^*a*^ of:	
		Daunorubicin	Hoechst 33342
Δ*efrCD*	EfrCD	32	2
	EfrCD_E512Q	2	0.25
Wild type	EfrCD	32	2
	EfrCD_E512Q	16	1

a MICs were determined in cation-supplemented Mueller-Hinton broth with 10 μg/ml erythromycin after 18 h at 37°C from a minimum of three independent experiments.

### EfrAB, EfrCD, and EfrEF mediate fluorescent dye efflux when overexpressed in Lactococcus lactis.

The analysis of the E. faecalis transporter gene deletion mutants does not take into account the gene expression levels of the ABC transporters under study. Previous studies on the transcriptional responses of E. faecalis V583 to chloramphenicol and erythromycin revealed that in the presence of drugs, only a subset of drug efflux pumps are upregulated (38, 39). For this reason, some of the transporters we investigated may not be expressed under our experimental conditions, which could explain why we did not observe a phenotype for the majority of the transporter gene deletions. To study the transporters independently of their expression in the endogenous context of E. faecalis, the seven enterococcal ABC exporters were overexpressed in L. lactis. E. faecalis and L. lactis both belong to the lactic acid bacteria and are phylogenetically closely related (40). In addition, well-established gene expression systems based on the nisin promoter exist for L. lactis (41), making this bacterium the ideal expression host for the transporters investigated. The heterodimeric ABC exporter LmrCD of L. lactis shares 28 to 59% sequence identity with the seven enterococcal ABC exporters (Table S1 in the supplemental material) and has been shown to transport the fluorescent dyes ethidium, Hoechst 33342, and 2′,7′-bis-(2-carboxyethyl)-5(6)-carboxyfluorescein acetoxymethyl ester (BCECF-AM) (30, 42). To exclude any masking of drug efflux by LmrCD, we performed the experiments in the L. lactis Δ*lmrA* Δ*lmrCD* strain (33). For functional analysis of the seven E. faecalis transporters, the conserved Walker B glutamate of the consensus site was mutated to a glutamine (E-to-Q mutant) and served as a negative control.

**FIG 3:**
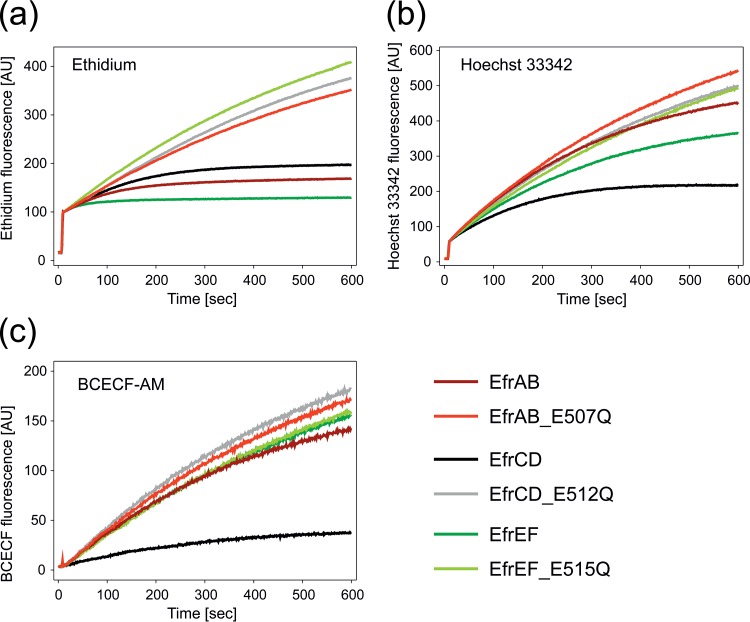
Fluorescent dye transport mediated by EfrAB, EfrCD, and EfrEF. Fluorescence spectroscopy was used to measure the accumulation of ethidium (a), Hoechst 33342 (b), and BCECF-AM (c) by L. lactis NZ9000 Δ*lmrA* Δ*lmrCD* cells expressing the respective wild-type or inactive E-to-Q mutant transporter. Active efflux manifests in a slower increase of fluorescence.

By use of washed and glucose-energized L. lactis NZ9000 Δ*lmrA* Δ*lmrCD* containing the overexpressed transporters, the accumulation of the fluorescent multidrug transporter substrates ethidium, Hoechst 33342, and BCECF-AM was monitored. Active efflux is observed when the increase in the fluorescent signal in cells expressing the wild-type transporter is slower than that in cells expressing the inactive E-to-Q mutant. EfrCD was the only transporter showing marked transport activity for all three dyes (Fig. 3). EfrAB and especially EfrEF exhibited strong ethidium transport and were also capable of Hoechst 33342 transport (Fig. 3). Interestingly, the other four transporters (EF0942/41, EF1592/93, EF1733/32, and EF2593/92) failed to exhibit measurable transport activity for these three typical multidrug efflux compounds (Fig. S1 in the supplemental material).

### Overexpression of EfrAB, EfrCD, and EfrEF in L. lactis confers multidrug resistance.

Further, we investigated whether overexpression of the transporters renders L. lactis NZ9000 Δ*lmrA* Δ*lmrCD* more resistant to drugs and dyes. Overexpression of the transporter EfrCD resulted in increased resistance to tetracycline, rifampin, daunorubicin, doxorubicin, acriflavine, ethidium, and Hoechst 33342 (Table 3). EfrAB and EfrEF exhibited drug efflux spectra similar to that of EfrCD but were also capable of exporting the fluoroquinolones ciprofloxacin (a 2-fold MIC increase was conferred by EfrEF), norfloxacin (a 1.5-fold MIC increase was conferred by EfrAB and a 2-fold MIC increase by EfrEF), and ofloxacin (2-fold MIC increases were conferred by EfrAB and EfrEF). EfrCD exhibited the strongest phenotype for multidrug transport, displaying 16-fold and 32-fold increases in the MICs of daunorubicin and doxorubicin, respectively. This is in agreement with the observations of the E. faecalis gene deletions and the transport activity measurements with fluorescent dyes in L. lactis. With the remaining four transporters, EF0942/41, EF1592/93, EF1733/32, and EF2593/92, no major MIC changes (≥2-fold) were observed for any compound tested. Of note, the 1.5-fold-increased MIC of tetracycline mediated by EF1733/32, although reproducibly and accurately measured, was not considered strong enough to justify the conclusion that this transporter is a drug efflux pump.

**TABLE 3:** Increase in drug resistance upon overexpression of enterococcal transporters in L. lactis

Drug tested	Fold change in MIC^*a*^ with overexpression of:						
	EfrAB	EfrCD	EfrEF	EF0942/41	EF1592/93	EF1733/32	EF2593/92
Ciprofloxacin	1	1	**2**	1	1	1	1
Norfloxacin^*b*^	**1.5**	1	**2**	1	1	1	1
Ofloxacin	**2**	1	**2**	1	1	1	1
Gentamicin	1	1	1	1	1	1	1
Kanamycin	1	1	1	1	1	1	1
Minocycline	1	1	1	1	1	1	1
Tetracycline^*b*^	**1.5**	**1.5**	1	1	1	**1.5**	1
Rifampin	1	**2**	1	1	1	1	1
Daunorubicin	**2**	**16**	**4**	1	1	1	1
Doxorubicin	**2**	**32**	**8**	1	1	1	1
Acriflavine	**2**	**2**	**4**	1	1	1	1
Ethidium	**4**	**4**	**4**	1	1	1	1
Hoechst 3334*2*^*b*^	**1.5**	**4**	**2**	1	1	1	1

a Calculated as the MIC for cells expressing the wild-type transporter divided by the MIC for cells expressing the inactive E-to-Q mutant. MICs were determined in GM17, 5 μg/ml chloramphenicol, and 1:5,000 (vol/vol) nisin after 16 h at 30°C from a minimum of three independent experiments.

b MICs were determined using a narrow series of drug concentrations, which allowed the measurement of MIC differences of <2-fold.

### Purification of the seven enterococcal ABC transporters.

**FIG 4:**
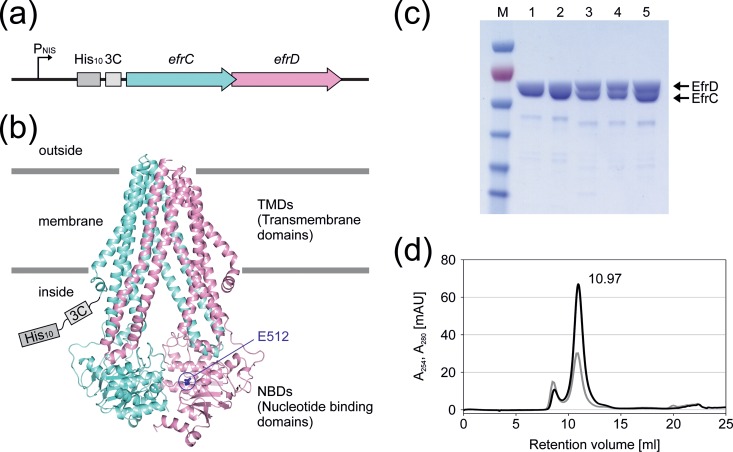
Expression and purification of EfrCD. (a) L. lactis expression construct containing the deca-His tag and 3C protease cleavage site followed by the ORFs encoding EfrC and EfrD. (b) Homology model of EfrCD based on the coordinates of TM287/288. EfrC is shown in aquamarine and EfrD in pink, and the conserved Walker B glutamate (E512) of the consensus site is highlighted as blue sticks. The deca-His tag and 3C protease cleavage site are attached to the N terminus of EfrC. (c) SDS-PAGE analysis of the different purification steps. Lanes: 1, Ni^2+^-NTA elution; 2, PD-10 elution; 3, after 3C protease cleavage; 4, after Ni^2+^-NTA rebinding; 5, main peak fraction of size exclusion chromatography separation. (d) Size exclusion chromatogram of EfrCD using a Superdex 200 Increase 10/300 GL column. *A*_280_ is shown in black and *A*_254_ in gray. The main peak, eluting at ca. 11 ml, corresponds to an EfrCD heterodimer.

Insufficient overexpression or protein aggregation in L. lactis NZ9000 Δ*lmrA* Δ*lmrCD* could explain why four out of seven transporters did not exhibit drug transport activities. To explore this possibility, the seven transporters were overexpressed in, and purified from, L. lactis via a deca-His tag attached to the N terminus of the first chain of the heterodimeric transporter complex. In agreement with previous studies on LmrCD (43), PatAB (25), and TM287/288 (20), the enterococcal transporters were purified as heterodimers, as judged from SDS-PAGE (Fig. 4; see also Fig. S2 in the supplemental material), indicating that a stable heterodimeric complex was formed between the corresponding half-transporters. The identities of the SDS-PAGE bands corresponding to EfrC and EfrD (Fig. 4c) were confirmed by matrix-assisted laser desorption ionization—time of flight (MALDI-TOF) analysis (not shown). Size exclusion chromatography analysis of the Ni^2+^-NTA-purified transporters revealed that they elute at a retention volume of ca. 11 ml, which corresponds to the size of a typical heterodimeric ABC exporter (44) (Fig. S2). Hence, without any exception, the transporter complexes appeared to be well folded and even withstood the protein purification procedure, including extraction from the membrane by detergent. This provides strong evidence that in the native context of the membrane, all transporters are correctly assembled, precluding protein aggregation as a cause for missing transport activity. For all transporters, an inactive E-to-Q mutant was purified and served as a negative control in the ATPase activity measurements for background subtraction. The expression levels of the E-to-Q mutants were equal to those of the wild-type transporters (not shown). A narrow fraction of the SEC peak eluting at 11 ml was used for the determination of basal ATPase activity. (Basal activity stands for the ATP turnover of an ABC transporter in the absence of added substrates.) Except for EF1592/93, all transporters exhibited basal ATPase activities when measured in the presence of 1 mM ATP at 30°C. However, the ATP hydrolysis rates observed differed markedly, ranging from 350 to 12,000 nmol P_i_/min/mg of protein (Table 4).

**TABLE 4:** Basal ATPase activities in detergent and proteoliposomes

Transporter	Basal activity (nmol P_i_/min/mg of protein) in:	
	Detergent	Proteoliposomes
EfrAB	3,689 ± 61	939 ± 76
EfrCD	2,329 ± 130	549 ± 66
EfrEF	805 ± 11	146 ± 14
EF0942/41	578 ± 9	1,663 ± 137
EF1592/93^*a*^	−0.3 ± 2.4	ND^*b*^
EF1733/32	348 ± 4	ND
EF2593/92	12,146 ± 536	ND

a This purified transporter had no measurable ATPase activity.

b ND, not determined.

### Reconstitution of EfrAB, EfrCD, and EfrEF into proteoliposomes.

**FIG 5:**
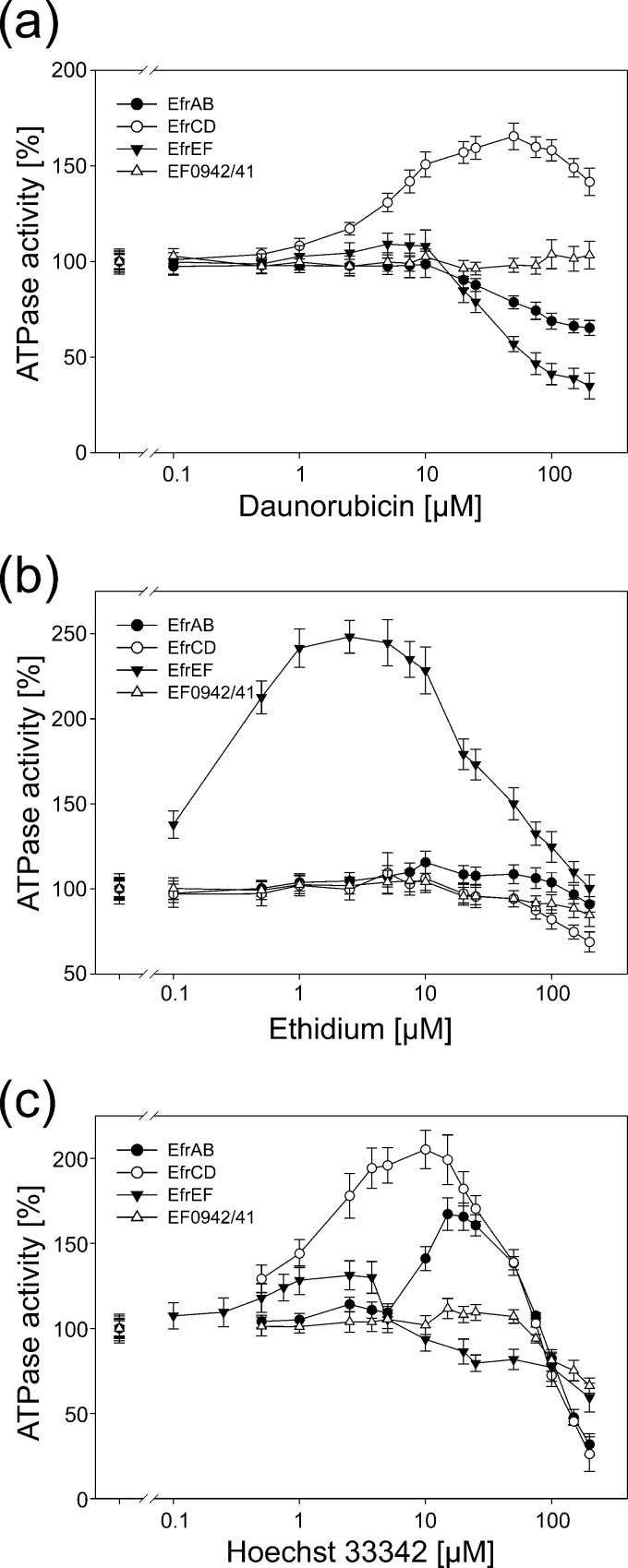
Drug-modulated ATPase activities of reconstituted enterococcal ABC exporters. The ATPase activities of EfrAB, EfrCD, EfrEF, and EF0942/41 were measured in the presence of daunorubicin (a), ethidium (b), and Hoechst 33342 (c) at drug concentrations ranging from 0.1 μM to 200 μM. ATPase activities were normalized to the basal activity of the respective transporter in the absence of drugs (set at 100%). The error bars correspond to the standard deviations for three technical replicates. The *x* axis has a logarithmic scale.

To investigate drug-induced modulation of the ATPase activity in a native environment, we reconstituted the four transporters EfrAB, EfrCD, EfrEF, and EF0942/41 (for each wild type and inactive E-to-Q mutant) into proteoliposomes. EF0942/41 was used as a control, because it can be well expressed and purified and exhibits basal ATPase activity but does not show multidrug efflux activity. Relative to those of detergent-purified proteins, the basal ATPase activities of reconstituted proteins were decreased for EfrAB, EfrCD, and EfrEF but were increased for EF0942/41 (Table 4). With the reconstituted transporters, drug-induced modulation of ATPase activity was measured in the presence of daunorubicin, ethidium, and Hoechst 33342 (Fig. 5). These drugs and dyes were chosen because they are all transported by EfrAB, EfrCD, and EfrEF. Multidrug ABC transporters respond in various ways to drug addition. There are reports of ATPase activation and inhibition (45, 46), as well as the occurrence of bell-shaped curves (47 – 49). In the majority of our measurements, we observed bell-shaped curves for the three multidrug transporters. ATPase inhibition as a result of drug addition was occasionally observed as well. Among the multidrug transporters, the magnitude of the responses differed greatly. Importantly, there were only minor modulations of ATPase activity for the control protein EF0942/41.

For daunorubicin, a strong response in the form of a bell-shaped curve was seen for EfrCD, whereas the ATPase activities of EfrAB and EfrEF declined markedly in the concentration range of 10 to 200 μM (Fig. 5a). The marked response for EfrCD correlates well with its capacity to transport this drug efficiently; upon EfrCD expression in L. lactis, the MIC of daunorubicin is increased 16-fold, while expression of EfrAB and EfrEF leads to MIC increases of 2- and 4-fold, respectively (Table 3). The control protein EF0942/41 did not respond at all, indicating that there is no nonspecific effect on the ATPase activities of ABC transporters in the daunorubicin concentration range sampled.

For ethidium, a bell-shaped curve with a large magnitude was observed for EfrEF, and the ATPase activity of EfrAB was modestly stimulated in the range of 10 to 100 μM (*P*, <0.05 for comparison by *t* test to the EF0942/41 control for all measurement points in this range) (Fig. 5b). In contrast, the ATPase activity of EfrCD was inhibited in the range of 100 to 200 μM (*P*, <0.05 for comparison to EF0942/41 by *t* test). The differences among the three multidrug transporters correlate well with the results of the transport experiments monitored by ethidium fluorescence (Fig. 3a), in which EfrEF exhibits the strongest and EfrCD the weakest ethidium transport activity. However, according to MIC determinations in L. lactis, the three multidrug transporters had equal abilities to confer ethidium resistance (Table 3).

For Hoechst 33342, all three multidrug transporters exhibited bell-shaped curves (Fig. 5c). In agreement with its pronounced transport phenotype in L. lactis as monitored by fluorescence and MIC determination, EfrCD exhibited distinct stimulation of its ATPase activity by Hoechst 33342 in proteoliposomes. Maximal stimulation of 165% for EfrAB was seen at a Hoechst 33342 concentration of 20 μM, while EfrEF had its maximum of 130% at 2.5 μM Hoechst 33342, indicating that EfrEF has a higher affinity for Hoechst 33342 than EfrAB. This difference in affinity may explain the slight differences observed between these transporters in the Hoechst 33342 transport assay based on fluorescence as well as in the resistance assays based on MIC determinations; in both transport assays, EfrEF transported Hoechst 33342 more efficiently than EfrAB. The ATPase activity of the control transporter EF0942/41 was not totally insensitive to the addition of Hoechst 33342. We observed a slight stimulation in the range of 20 to 50 μM and a distinct decrease in activity at 150 μM and 200 μM Hoechst 33342. EF0942/41, therefore, may recognize and transport Hoechst 33342 to some extent, but not sufficiently well to generate an observable transport phenotype. Alternatively, Hoechst 33342 may have intercalated into the lipid bilayer of the proteoliposome and in this manner indirectly influenced the properties of the transporter by changing the fluidity of the membrane.

It should be noted that with detergent-purified transporters, the ATPase activity was never seen to be stimulated, but was only inhibited, in the presence of transporter substrates (not shown). This indicates that drug modulation experiments need to be carried out in the context of a native lipid bilayer.

## DISCUSSION

Can multidrug transporters be identified on the basis of their sequences? In this study, we addressed this fundamental question by characterizing seven heterodimeric ABC exporters of E. faecalis. According to their entries in the National Center for Biotechnology Information (NCBI) database, these enterococcal transporters are all predicted to function as “ABC-type multidrug transport systems.” One of them, EfrAB, was previously described as a multidrug efflux pump when overexpressed in E. coli (12).

We thoroughly investigated the transporters in three complementary experimental settings. First, gene deletions in E. faecalis permitted us to study the transporters' contributions to drug efflux in the native context of this pathogenic bacterium. Second, overexpression in L. lactis, a closely related cousin of E. faecalis, permitted us to study the drug efflux capacities of the transporters independently of gene regulation in the native host. Third, biochemical experiments using purified and membrane-reconstituted transporters offered clues about the coupling between the transport substrate binding and ATPase activities of the transporters.

Our analysis revealed that only three of the seven predicted ABC multidrug transporters transported dyes and drugs that are recognized by typical drug efflux pumps. This finding, however, does not exclude the possibility that the remaining four transporters transport drugs that were not part of our screen. Under our experimental conditions, we found that the product of the *ef0789*–*ef0790* genes was the major multidrug efflux pump in E. faecalis among the set of ABC transporters investigated, and we named it EfrCD. The strong efflux activity and broad substrate spectrum of EfrCD were confirmed in L. lactis, and the ATPase activity of the purified and reconstituted transporter is robustly modulated by the drugs it transports. EfrCD is phylogenetically more closely related to L. lactis LmrCD (58.6% identity) and S. pneumoniae PatAB (57.4% identity) than to any of the other six enterococcal ABC transporters investigated in this study (Fig. 1; also Table S1 in the supplemental material). It is therefore not surprising that the substrate spectrum of EfrCD closely resembles that of LmrCD, which has been shown to confer strong resistance to daunorubicin and exhibits robust transport of the fluorescent dyes ethidium, Hoechst 33342, and BCECF-AM (30, 43). PatAB has been shown to transport acriflavine and ethidium, but in contrast to EfrCD, it also confers resistance to the fluoroquinolones ciprofloxacin and norfloxacin (24, 50).

EfrAB was described previously as a drug efflux pump expelling multiple dyes and drugs when overexpressed in E. coli (12). Interestingly, we found that deletion of the *efrAB* genes in E. faecalis had only minor consequences for the drug susceptibility profile, affecting resistance to acriflavine and ethidium in the E. faecalis strain 4205. In agreement with the findings of Lee et al. (12), EfrAB transported a large set of drugs upon heterologous overexpression, including doxorubicin, daunorubicin, acriflavine, ethidium, and norfloxacin. These observations suggest that EfrAB is poorly expressed in its native host, E. faecalis, but has the capacity to pump drugs when overexpressed from a plasmid. In analogy, the expression of EfrEF must be low in E. faecalis under our experimental conditions, because the corresponding gene deletion results only in 2-fold-decreased resistance to one of the drugs tested, namely, acriflavine. When overexpressed in L. lactis, EfrEF exhibited a drug efflux profile very similar to that of EfrAB, with which it shares a sequence identity of 38.9%. EfrEF was the only transporter exhibiting a drug efflux phenotype for ciprofloxacin, norfloxacin, and ofloxacin, and it potentially confers resistance to these fluoroquinolones when derepressed in E. faecalis by mutations.

The remaining four transporters—EF0942/41, EF1592/93, EF1733/32, and EF2593/92—were unable to transport any of the drugs included in this study, even when overexpressed in L. lactis (an exception is a modest, but reproducibly measured, 1.5-fold-increased MIC of tetracycline with EF1733/32). We showed that all transporters can be purified as heterodimers in detergent after overexpression in L. lactis. Therefore, low expression and/or protein aggregation can be excluded as reasons for lacking multidrug transport activity. Moreover, except for EF1592/93, the purified transporters exhibited basal ATPase activity, providing clear evidence that they undergo the conformational cycling required for transport. The missing ATPase activity of EF1592/93 may be explained by inactivation of the transporter in the presence of detergent or the lack of ATPase activity in the absence of the transport substrate. It is well known that the peptide transporter TAP1/2 strictly requires peptide binding to the TMDs to trigger ATP hydrolysis (51).

Our observations suggest that EF0942/41, EF1733/32, and EF2593/92 (and probably also EF1592/93) likely do not operate as multidrug efflux pumps, although that is their annotated function. In agreement with its lack of a drug efflux phenotype, the ATPase activity of reconstituted EF0942/41 is not modulated by daunorubicin, ethidium, or Hoechst 33342. In contrast, the ATPase activities of the verified multidrug transporters EfrAB, EfrCD, and EfrEF are modulated by their transport substrates daunorubicin, ethidium, and Hoechst 33342, indicating specific coupling between the drug binding site at the TMDs and ATP hydrolysis at the NBDs. Drug-induced ATPase modulation profiles manifested mainly as bell-shaped curves. These curves have been interpreted previously as arising from a sum of stimulating and inhibiting effects of drug addition. At low drug concentrations, ATPase activity is stimulated due to drug binding to its high-affinity site. At higher drug concentrations, ATPase activity decreases again due to the presence of a low-affinity drug release site, which becomes occupied at elevated drug concentrations, leading to inhibition of the transport cycle and ATPase activity (47). We also observed purely inhibitory curves, as, for example, for EfrAB in the presence of daunorubicin. In light of their strong correlation with transport, the ATPase activities of the reconstituted ABC transporters represent an excellent experimental readout for identification of further drug substrates of EfrAB, EfrCD, and EfrEF.

In summary, our analysis revealed two novel ABC multidrug transporters of E. faecalis, which—in analogy to the previously identified transporter EfrAB—were called EfrCD and EfrEF. Among these three transporters, EfrCD plays the most prominent role in the native context of E. faecalis, presumably because its protein production level is highest. On the basis of extensive biochemical experiments, four of seven ABC transporters appear to be unable to translocate the typical multidrug efflux transporter drugs included in this study, although they are annotated according to their protein sequences as “ABC-type multidrug transport systems.” It is well documented that multidrug transporters also recognize endogenous substrates and thereby fulfill physiological functions in the cell (52). It is therefore likely that the seven transporters recognize substrates other than dyes and drugs and that a subset—EfrAB, EfrCD, and EfrEF—are, in addition, capable of extruding drugs. Our analysis provides an excellent starting point for the identification of critical differences between closely related transporters, of which only a fraction appear to be capable of multidrug transport. Molecular hallmarks of drug efflux pumps are likely to be determined in future studies and will permit reliable prediction of drug efflux pumps based on their amino acid sequences.

## Supplementary Material

Supplemental material
