# Supplementary material for: The Heterodimeric ABC Transporter EfrCD Mediates Multidrug Efflux in Enterococcus faecalis
Source: Antimicrob Agents Chemother. 2016 Aug 22;60(9):5400–11. doi: 10.1128/AAC.00661-16 (PMC4997860; doi:10.1128/AAC.00661-16)
Supplement: Supplemental material [file supp_60_9_5400__index.html]

The Heterodimeric ABC Transporter EfrCD Mediates Multidrug Efflux in Enterococcus faecalis — Supplemental material 

# The Heterodimeric ABC Transporter EfrCD Mediates Multidrug Efflux in Enterococcus faecalis

## Supplemental material

- Supplemental file 1 -

  Tables S1-S4 and Fig. S1-S4

  PDF, 2.1M
