# Supplementary material for: The Heterodimeric ABC Transporter EfrCD Mediates Multidrug Efflux in Enterococcus faecalis
Source: Antimicrob Agents Chemother. 2016 Aug 22;60(9):5400–11. doi: 10.1128/AAC.00661-16 (PMC4997860; doi:10.1128/AAC.00661-16)
Supplement: Supplemental material [file AAC.00661-16_zac009165484so1.pdf]

TABLE S1: Sequence identity matrix of the seven ABC exporters of *E. faecalis* V583, LmrCD of *L. lactis* IL1406, PatAB of *S. pneumoniae* R6, and TM287/288 of *T. maritima* MSB8.

	Identity[a]									
	LmrCD	PatAB	TM287/288	EfrAB[b]	EfrCD[c]	EfrEF[d]	EF0942/41	EF1592/93	EF1733/32	EF2593/92
LmrCD	100.0	57.0	36.1	34.0	58.6	32.2	31.8	30.9	28.5	34.1
PatAB	57.0	100.0	37.2	35.9	57.4	31.3	32.3	33.5	27.1	33.8
TM287/288	36.1	37.2	100.0	38.2	38.8	35.4	39.5	35.9	29.9	40.4
EfrAB[b]	34.0	35.9	38.2	100.0	37.5	38.9	37.1	31.4	29.6	42.9
EfrCD[c]	58.6	57.4	38.8	37.5	100.0	32.9	33.7	31.5	29.1	35.0
EfrEF[d]	32.2	31.3	35.4	38.9	32.9	100.0	36.0	29.0	27.3	39.9
EF0942/41	31.8	32.3	39..5	37.1	33.7	36.0	100.0	29.2	29.8	41.1
EF1592/93	30.9	33.5	35.9	31.4	31.5	29.0	29.2	100.0	32.9	31.3
EF1733/32	28.5	27.1	29.9	29.6	29.1	27.3	29.8	32.9	100.0	29.4
EF2593/92	34.1	33.8	40.4	42.9	35.0	39.9	41.1	31.3	29.4	100.0

[a] Sequence identity matrix was generated using ClustalW

[b] EfrAB corresponds to EF2920/19

[c] EfrCD corresponds to EF0789/90

[d] EfrEF corresponds to EF2226/27

TABLE S2: Sequences of PCR primers for the generation of gene deletion knockouts in *E .faecalis*.

efrAB (*ef2920/19*)	5'-FW: atatatGCTCTTCtagtCCAGGTCCGCAATCCTGCTTC<br>5'-RV: gtattaccgatttcGAATAAAAAGAGGTCGGGACAGAAGCG<br>3'-FW: cccgacctctttttattcGAAATCGGTAATACAAGCTGTCTGGCTAG<br>3'-RV: tatataGCTCTTCatgcGTGACCGTGGGTAATAACGAGTGC
efrCD (*ef0789/90*)	5'-FW: atatatGCTCTTCtagtGACTGGGTGTTCTGGGATGAATGG<br>5'-RV: gccgtatattcctcCCTGCTTCTTCAGTGTTGGTCTGC<br>3'-FW: ctgaagaagcaggGAGGAATATACGGCAATTACGGCGC<br>3'-RV: tatataGCTCTTCatgcGAGCTAAACCGTTCGCTAAACCTGAC
efrEF (*ef2226/27*)	5'-FW: atatatGCTCTTCtagtGCTTAGCTACATTTGCTATTGCTAGTTCCT<br>5'-RV: gtcggagcttGGGTTCCTGCGATTTGCAAACAG<br>3'-FW: gcaggaacccAAGCTCCGACTCTTTACGCCAG<br>3'-RV: tatataGCTCTTCatgcCGTCCAACTGGTAAATTGCACCTAGGAC
ef0942/41	5'-FW: atatatGCTCTTCtagtCCGCAGCGACTGGAAGTCC<br>5'-RV: aataaccctgttcGGGCAAGACTAACTCGAAACAAGCTTC<br>3'-FW: gttagtcttgcccGAACAGGGTTATTATCGTGACCTTTACGAAGC<br>3'-RV: tatataGCTCTTCatgcCTTTAGCGACCAATATTGGCAGTGCTG
ef1592/93	5'-FW: atatatGCTCTTCtagtGGTGTTTGGGAACATAATCATCCAGCG<br>5'-RV: agttgatcatgagCGACAAAAATAACAGCTAGCAGTAACTTATTC<br>3'-FW: gctgttatttttgtcgCTCATGATCAACTAATGGCAAAACATGCG<br>3'-RV: tatataGCTCTTCatgcCCGTCAGTCATTGAGTCTGCGAC
ef1733/32	5'-FW: atatatGCTCTTCtagtCGAAGTCTTGTGGAAAGAGCAAATCCC<br>5'-RV: cataaaggccacccGCTCTTTTCCTCCTAGATTATCCTATTCTCCC<br>3'-FW: gaggaaaagagcgGGTGGCCTTTATGCGGATATGTATCAG<br>3'-RV: tatataGCTCTTCatgcCCTAAACTTTCAGCGACACTTTCTGCAC
ef2593/92	5'-FW: atatatGCTCTTCtagtCGTTTCGGCGAGTCCGTCATTCC<br>5'-RV: cctcatcatgaattacCTCCTCCTTTTTGCTACTTATAAGCGCC<br>3'-FW: gcaaaaaggaggagGTAATTCATGATGAGGCTTCAGGAACCATAGTC<br>3'-RV: tatataGCTCTTCatgcGGTGGGTTTTACTTGTGCCAGTCC

TABLE S3: Sequences of PCR primers for the generation of FX-compatible knockout and complementation vectors.

pCJK245_FX	
ccdB from pINIT_cat	ccdB_XbaI_FW: atatatctagaGGCCGCTCTTCTAGTCGACCTG<br>ccdB_NcoI_RV: atatatccatggCGGTGGCCGCTCTTCATGC
pMSP3535_FX_em and pMSP3535_FX_cat	
pMSP3535_FX_em	pMSP3535_FX_FW: CGCTCTTCCGCaTaaTCGCTCACTGAC<br>pMSP3535_FX_RV: atatatgctcttcTACTCATTTTGAGTGCCTCCTTATAATTTATTTTGTAG
pMSP3535_FX_cat	pMSP3535_FX_cat_FW: CGAACACGAACCGTCTTATCTCCCATTATATC<br>pMSP3535_FX_cat_RV: GGAGAAACTTGGAACTAGCATTTAGAGAAAGC
cat from pCJK245	cat_FW: GCGAACGAAAAACAATTGCAAAAGCAGATTG<br>cat_RV: GCACACGAAAAACAAGTTAAGGGATGC

TABLE S4: Sequences of PCR primers for amplification of the open reading frames from genomic DNA of *E. faecalis* V583 and for the generation of the inactive E to Q mutant by mutating the conserved Walker B glutamate of the consensus site to a glutamine using QuikChange site-directed mutagenesis.

	Amplification of ORF from gDNA	QuikChange of E to Q mutant
efrAB (ef2920/19)	FW: atatatGCTCTTCtagtAAGTTAATGTGGCGTTACACAATGCG<br>RV: tatataGCTCTTCatgcTTCCTCATAGTCGCCTTCTTCAGC	FW: TTGATTTTAGATcAGGCGACTAGTTCAGTCG<br>RV: CGACTGAACTAGTCGCCTgATCTAAAATCAA
efrCD (ef0789/90)	FW: atatatGCTCTTCtagtGGTTCCGGCGGCGGTGGCGGTTCTGACCTTATTATTCAACACGCC<br>RV: tatataGCTCTTCatgcTTCAAAAACAAATTGATTTTTATAAAGTTCCGC	FW: GAATTATTAATTTTGGATcAAGCAACAAG<br>RV: CTTGTTGCTTgATCCAAAATTAATAATTC
efrCD (ef0789/90) with its native promoter	FW: atatatGCTCTTCtagtCACTTTATGATAGGAAAAACTTGTGAAAAATTAACTTGTTCG<br>RV: tatataGCTCTTCatgcTTCAAAAACAAATTGATTTTTATAAAGTTCCGC	FW: GAATTATTAATTTTGGATcAAGCAACAAG<br>RV: CTTGTTGCTTgATCCAAAATTAATAATTC
efrEF (ef2226/27)	FW: atatatGCTCTTCtagtAAATTAATGAAAGAGTTTATTAAAGAAAATAAATGGATTGTTCTTGCG<br>RV: tatataGCTCTTCatgcAGTGGTTTGAAATTGACTATTATATAAGCTGGCG	FW: CTCGATcAAGCAACTTCGAGTGTGGAC<br>RV: CGAAGTTGCTTgATCGAGAATAACAACGGG
ef0942/41	FW: atatatGCTCTTCtagtTTTGGTTTATTGAAATACGCAAAAAATTATCGAAAACAAA<br>RV: tatataGCTCTTCatgcTTGTGCTTTTTGATTAAACTGTGCTTCGTAAAG	FW: GATGCTTATTTTAGATcAAGCGACCAGTTCAG<br>RV: CTGAACTGGTCGCTTgATCTAAAATAAGCATC
ef1592/93	FW: atatatGCTCTTCtagtAATAGTTTTGAATGGATTTGGCAATATGCTAAAA<br>RV: tatataGCTCTTCatgcGCTCTTTAATAAGTCGTATTGCGATTGATATAAATG	FW: ATTTTAATTTTAGACcAAGCAACCTCCAGTAT<br>RV: ATACTGGAGGTTGCTTgGTCTAAAATTAAAAT
ef1733/32	FW: atatatGCTCTTCtagtTCCATATTCAAAAAATTAGGCTGGTTTTTTAAGC<br>RV: tatataGCTCTTCatgcAACTTCCGTACTTTGTAACTGATACATATCCGC	FW: CCAAAATTTTAGTTTTAGATcAAGCAACCGCTAATATT<br>RV: ATATTAGCGGTTGCTTgATCTAAAACTAAAATTTTGG
ef2593/92	FW: atatatGCTCTTCtagtATTAAACTAGTCAAACGGATGTCATTATGGTCGG<br>RV: tatataGCTCTTCatgcGGCTACTTCTTCTGAAAACTGACTGTTATATAAGTCAG	FW: CGTTTTAATTTTGGATcAAGCCACCTCAAGTG<br>RV: CACTTGAGGTGGCTTgATCCAAAATTAAAACG

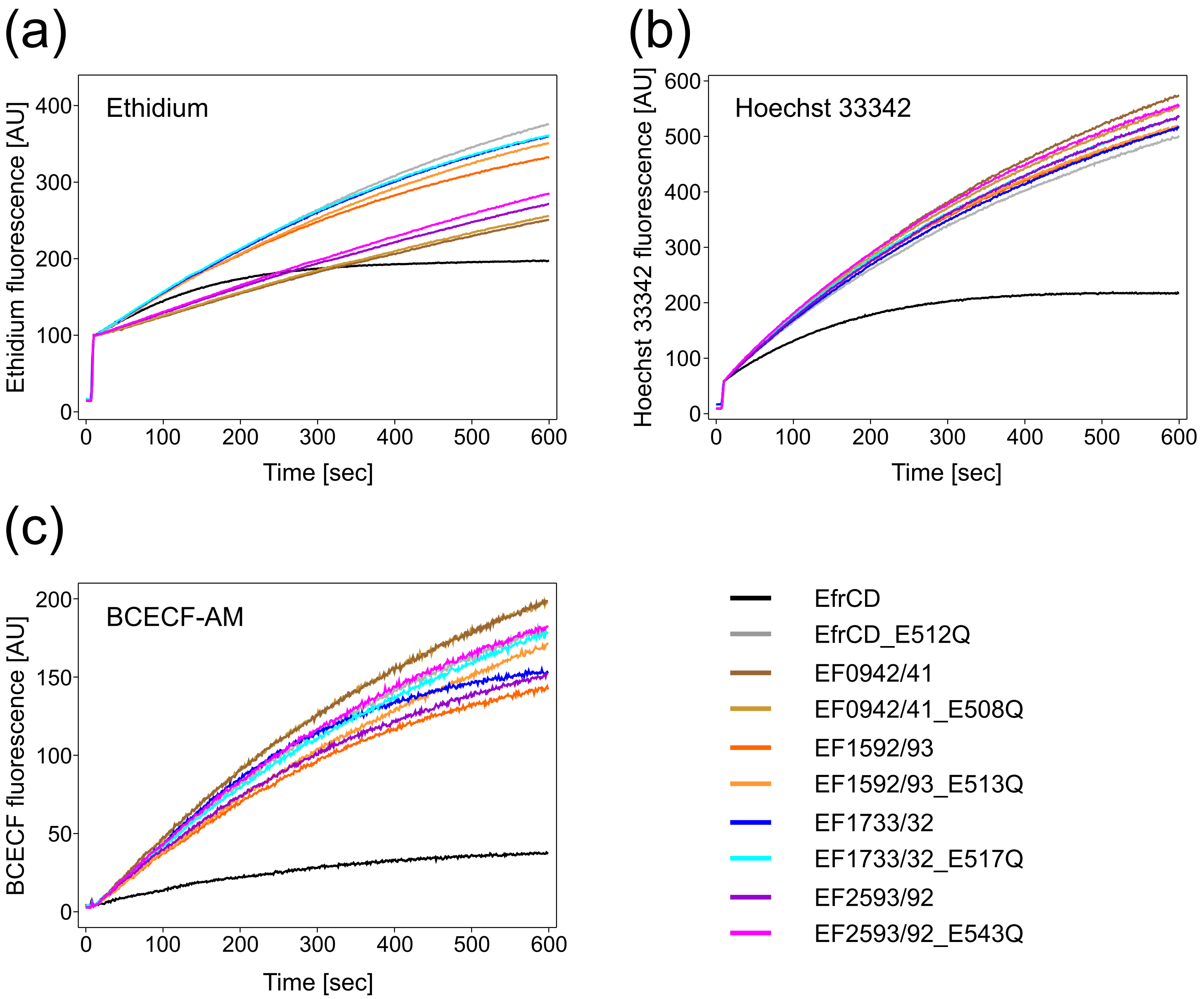

Supplementary Figure 1

Fluorescent dye transport mediated by EF0942/41, EF1592/93, EF1733/32 and EF2593/92. Ethidium (a), Hoechst 33342 (b) and BCECF-AM (c) transport was measured as shown in Figure 1. *L. lactis* NZ9000 *ΔlmrAΔlmrCD* cells expressing wildtype or inactive E to Q mutant transporters of EF0942/41, EF1592/93, EF1733/32 and EF2593/92 were supplemented with ethidium, Hoechst 33342, or BCECF-AM and fluorescence was measured. The traces for EfrCD shown in Figure 1 are plotted for reference.

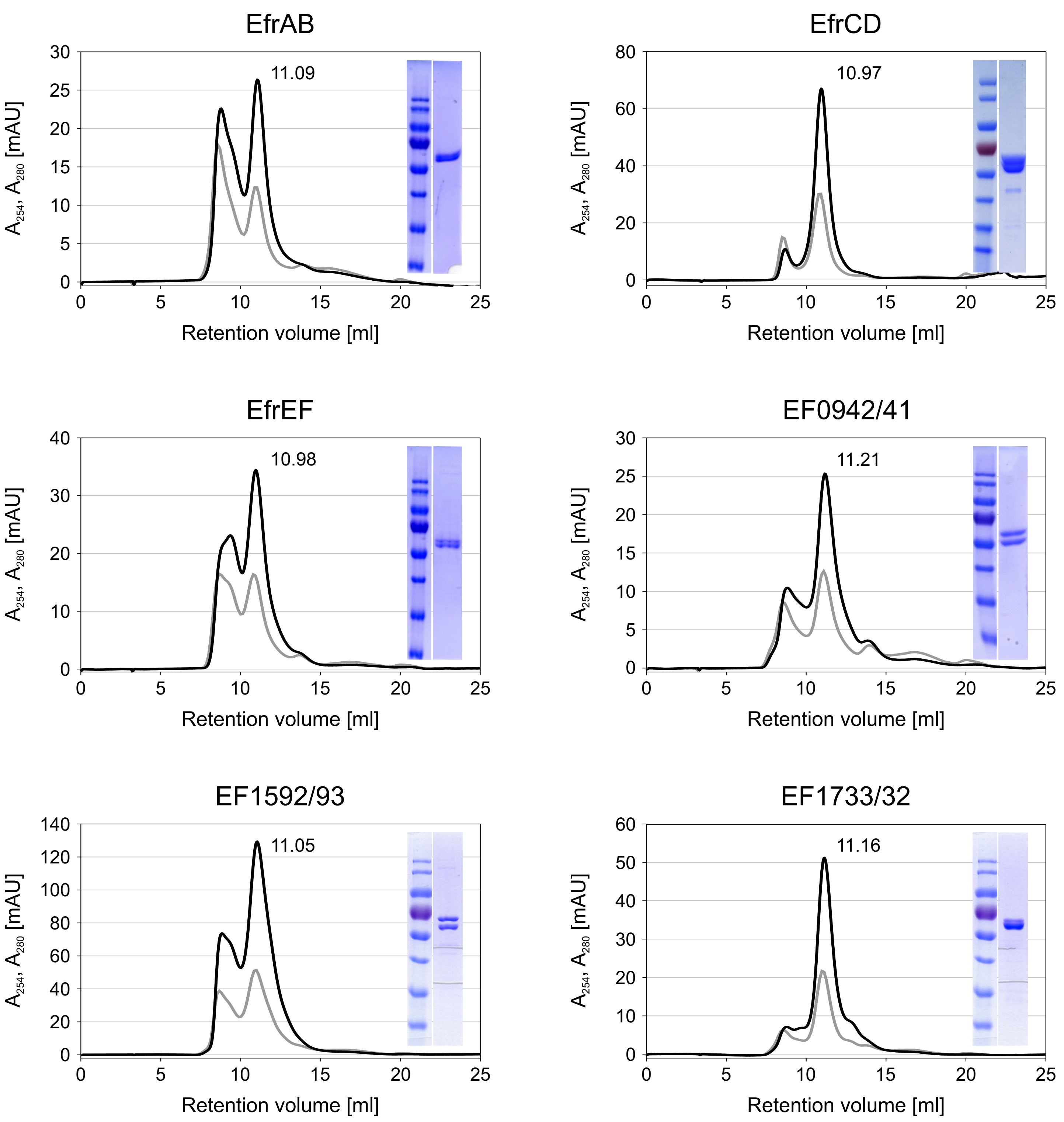

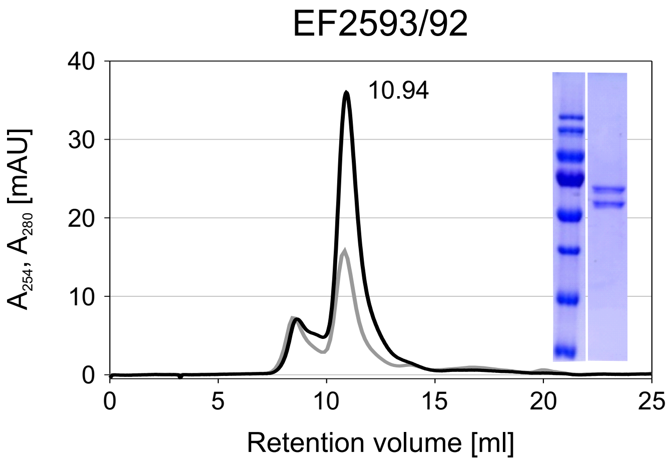

[μg of protein/ liter of expression culture]	
EfrAB	11
EfrCD	60
EfrEF	16
EF0942/41	11
EF1592/93	64
EF1733/32	24
EF2593/92	16

Supplementary Figure 2

SEC profiles of purified enterococcal ABC exporters. The proteins were expressed in and purified from *L. lactis*. Size-exclusion chromatography using a Superdex 200 increase 10/300 GL column was performed in 20 mM Tris/HCl pH 7.4, 150 mM NaCl and 0.03% β-DDM. The peak eluting at around 11 ml retention volume corresponds to the size of typical heterodimeric ABC exporters. A_{280} is shown in black and A_{254} in gray. Peak fractions used for ATPase activity measurements were analyzed by SDS-PAGE and shown as insets. The protein yields contained in the respective 11 ml peak fractions (0.5 ml) were normalized to µg/liter of expression culture and listed in the table.

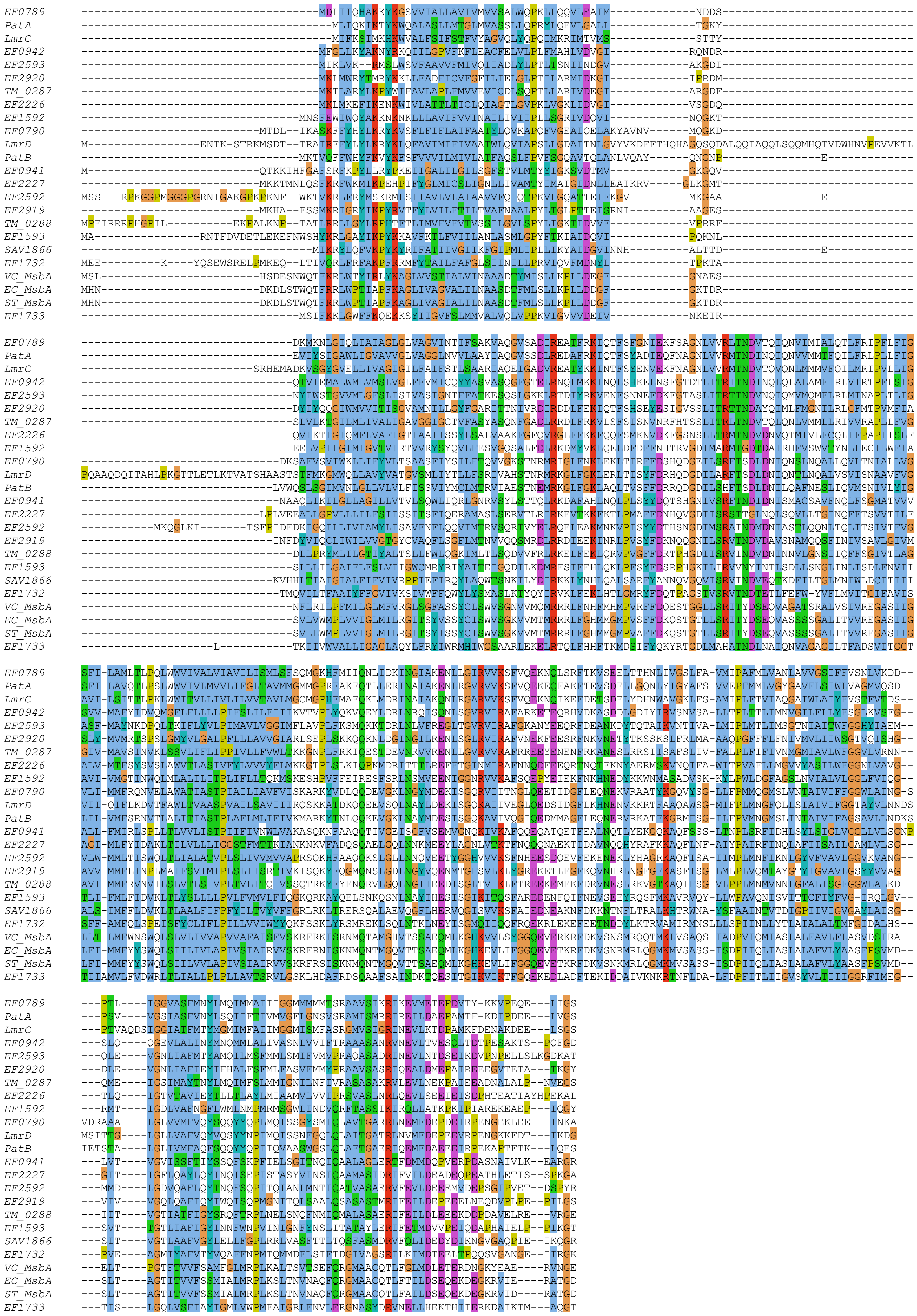
EF0789
PatA
LmrC
EF0942
EF2593
EF2920
TM_0287
EF2226
EF1592
EF0790
LmrD
PatB
EF0941
EF2227
EF2592
EF2919
TM_0288
EF1593
SAV1866
EF1732
VC_MsbA
EC_MsbA
ST_MsbA
EF1733

Supplementary Figure 3

Alignment of TMDs for homology model of EfrCD. Transmembrane domains (TMDs) of bacterial ABC exporters were aligned using MAFFT for model building.

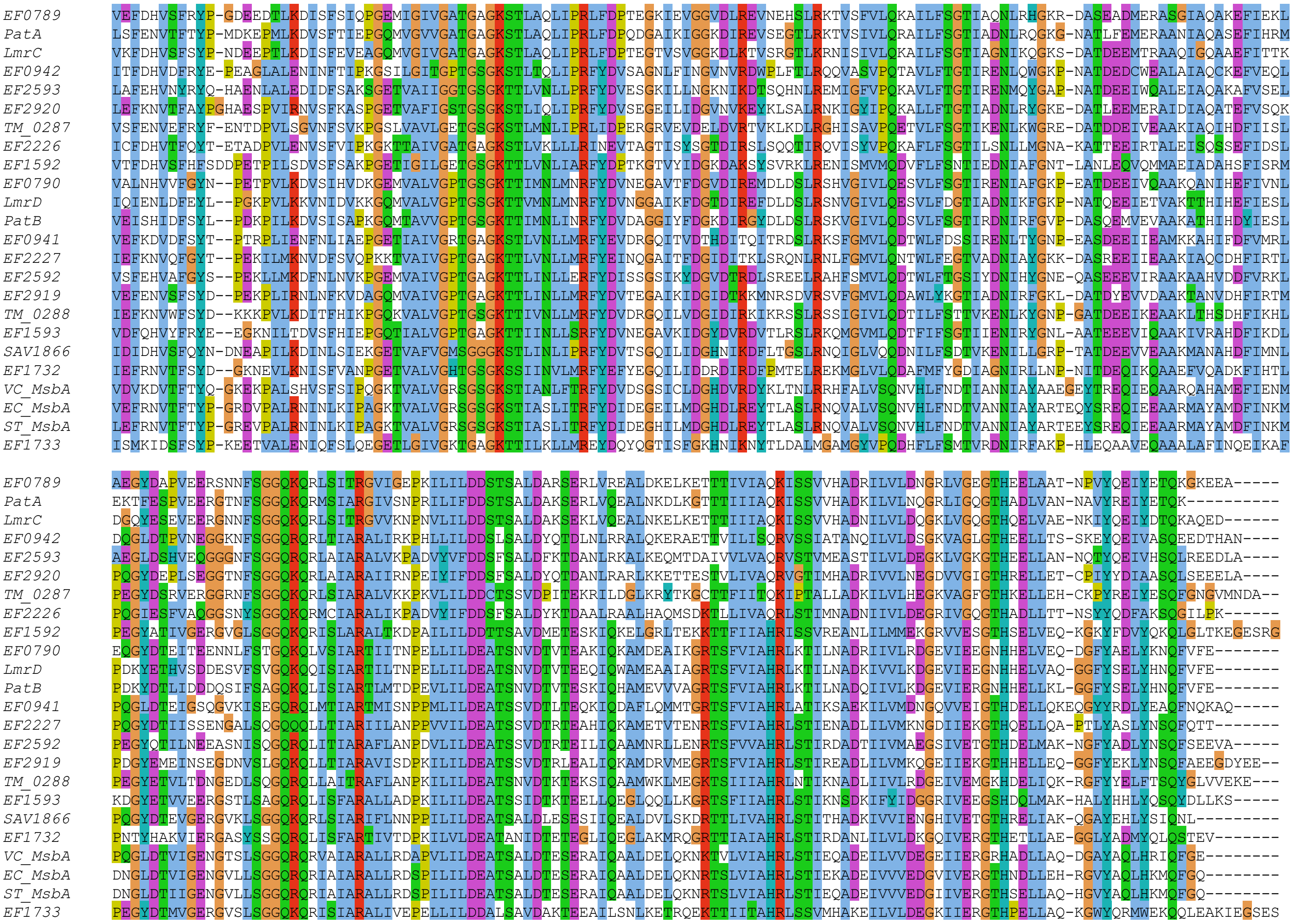

Supplementary Figure 4

Alignment of NBDs for homology model of EfrCD. Nucleotide binding domains (NBDs) of bacterial ABC exporters were aligned using MAFFT for model building.
